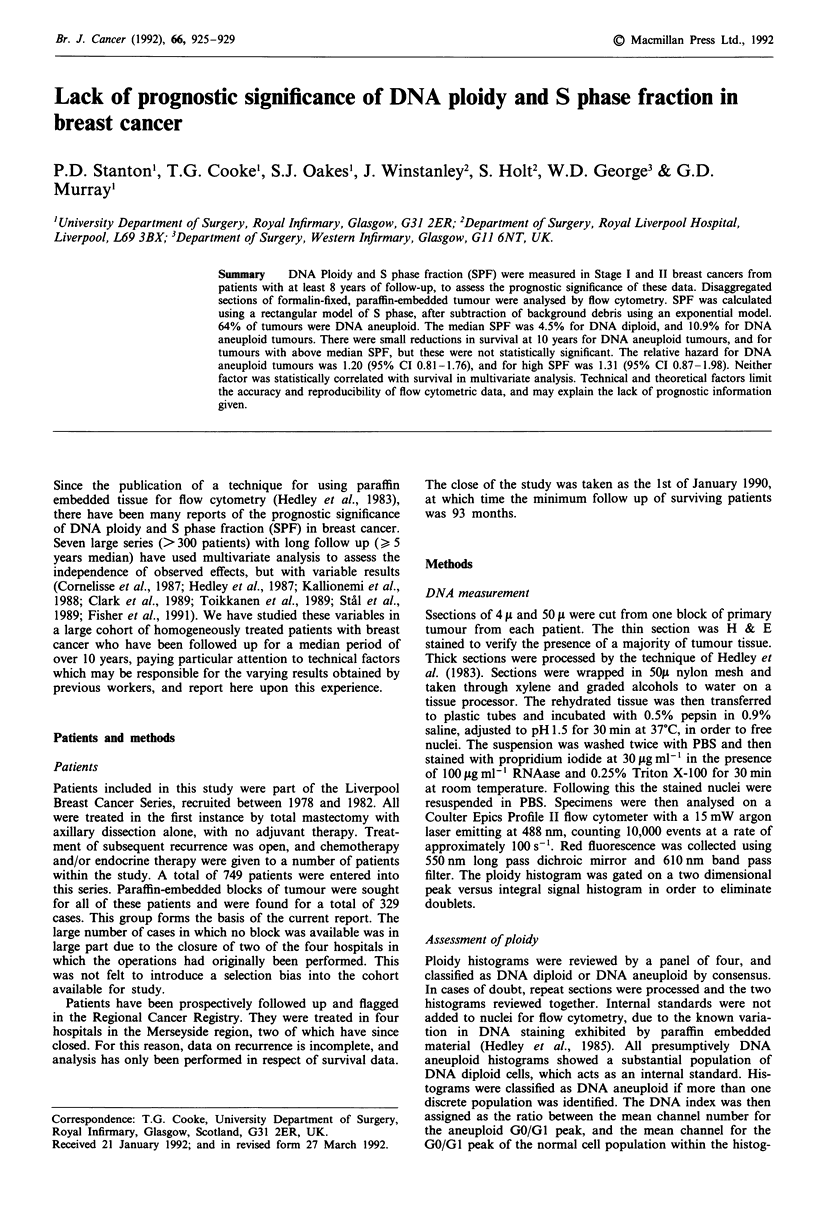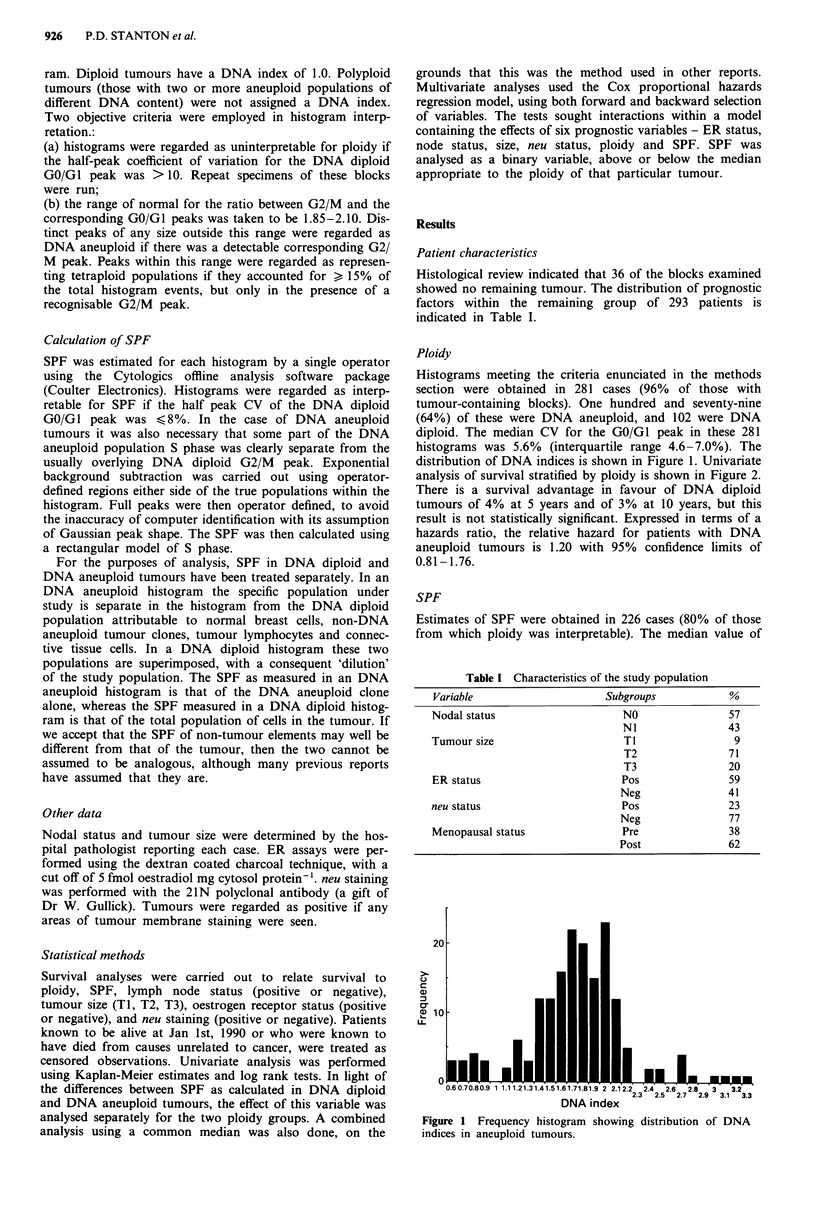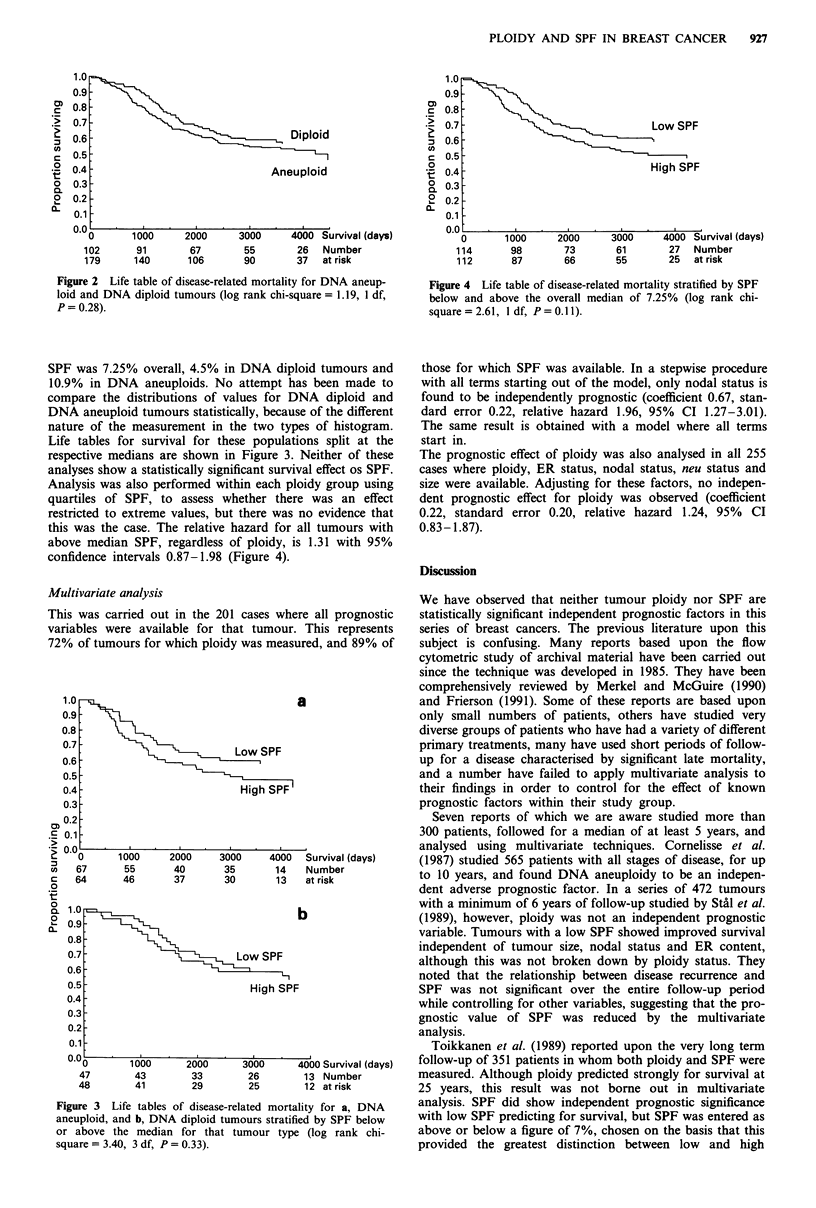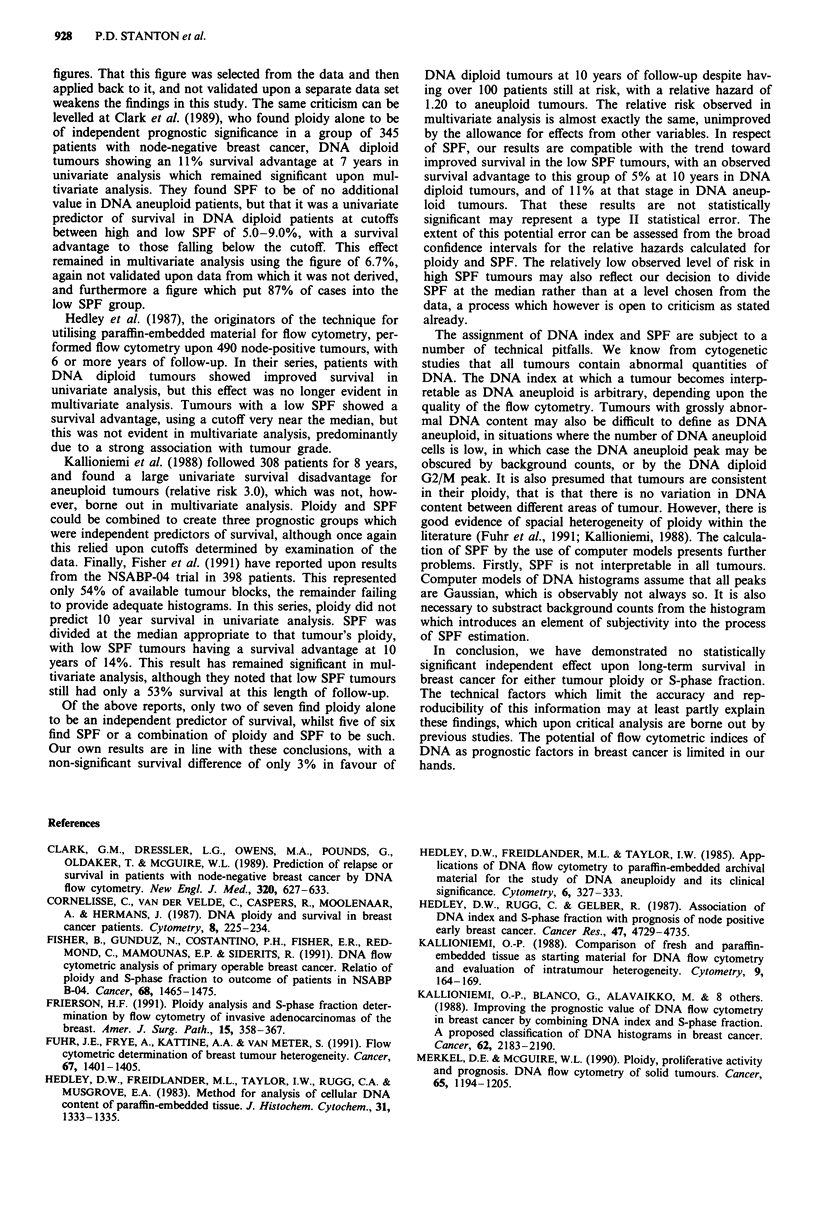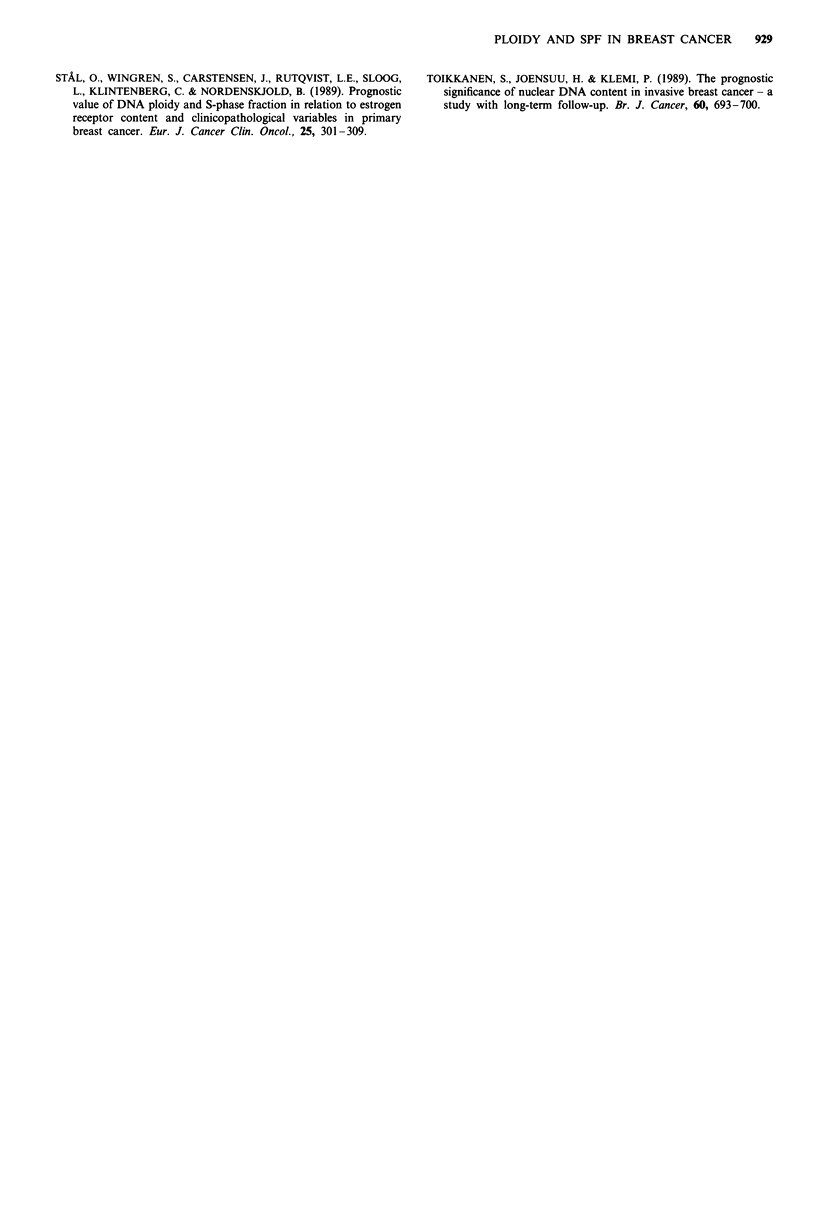# Lack of prognostic significance of DNA ploidy and S phase fraction in breast cancer.

**DOI:** 10.1038/bjc.1992.387

**Published:** 1992-11

**Authors:** P. D. Stanton, T. G. Cooke, S. J. Oakes, J. Winstanley, S. Holt, W. D. George, G. D. Murray

**Affiliations:** University Department of Surgery, Royal Infirmary, Glasgow, Scottland, UK.

## Abstract

DNA Ploidy and S phase fraction (SPF) were measured in Stage I and II breast cancers from patients with at least 8 years of follow-up, to assess the prognostic significance of these data. Disaggregated sections of formalin-fixed, paraffin-embedded tumour were analysed by flow cytometry. SPF was calculated using a rectangular model of S phase, after subtraction of background debris using an exponential model. 64% of tumours were DNA aneuploid. The median SPF was 4.5% for DNA diploid, and 10.9% for DNA aneuploid tumours. There were small reductions in survival at 10 years for DNA aneuploid tumours, and for tumours with above median SPF, but these were not statistically significant. The relative hazard for DNA aneuploid tumours was 1.20 (95% CI 0.81-1.76), and for high SPF was 1.31 (95% CI 0.87-1.98). Neither factor was statistically correlated with survival in multivariate analysis. Technical and theoretical factors limit the accuracy and reproducibility of flow cytometric data, and may explain the lack of prognostic information given.


### Lack of prognostic significance of DNA ploidy and S phase fraction in breast cancer

P.D. Stanton[1], T.G. Cooke[1], S.J. Oakes[1], J. Winstanley[2], S. Holt[2], W.D. George[3] & G.D. Murray[1]

*[1]University Department of Surgery, Royal Infirmary, Glasgow, G31 2ER; [2]Department of Surgery, Royal Liverpool Hospital, Liverpool, L69 3BX; [3]Department of Surgery, Western Infirmary, Glasgow, G11 6NT, UK.*

**Summary** DNA Ploidy and S phase fraction (SPF) were measured in Stage I and II breast cancers from patients with at least 8 years of follow-up, to assess the prognostic significance of these data. Disaggregated sections of formalin-fixed, paraffin-embedded tumour were analysed by flow cytometry. SPF was calculated using a rectangular model of S phase, after subtraction of background debris using an exponential model. 64% of tumours were DNA aneuploid. The median SPF was 4.5% for DNA diploid, and 10.9% for DNA aneuploid tumours. There were small reductions in survival at 10 years for DNA aneuploid tumours, and for tumours with above median SPF, but these were not statistically significant. The relative hazard for DNA aneuploid tumours was 1.20 (95% CI 0.81-1.76), and for high SPF was 1.31 (95% CI 0.87-1.98). Neither factor was statistically correlated with survival in multivariate analysis. Technical and theoretical factors limit the accuracy and reproducibility of flow cytometric data, and may explain the lack of prognostic information given.

Since the publication of a technique for using paraffin embedded tissue for flow cytometry (Hedley *et al.*, 1983), there have been many reports of the prognostic significance of DNA ploidy and S phase fraction (SPF) in breast cancer. Seven large series (>300 patients) with long follow up (⩾5 years median) have used multivariate analysis to assess the independence of observed effects, but with variable results (Cornelisse *et al.*, 1987; Hedley *et al.*, 1987; Kallionemi *et al.*, 1988; Clark *et al.*, 1989; Toikkanen *et al.*, 1989; Stål *et al.*, 1989; Fisher *et al.*, 1991). We have studied these variables in a large cohort of homogeneously treated patients with breast cancer who have been followed up for a median period of over 10 years, paying particular attention to technical factors which may be responsible for the varying results obtained by previous workers, and report here upon this experience.

### Patients and methods

### *Patients*

Patients included in this study were part of the Liverpool Breast Cancer Series, recruited between 1978 and 1982. All were treated in the first instance by total mastectomy with axillary dissection alone, with no adjuvant therapy. Treatment of subsequent recurrence was open, and chemotherapy and/or endocrine therapy were given to a number of patients within the study. A total of 749 patients were entered into this series. Paraffin-embedded blocks of tumour were sought for all of these patients and were found for a total of 329 cases. This group forms the basis of the current report. The large number of cases in which no block was available was in large part due to the closure of two of the four hospitals in which the operations had originally been performed. This was not felt to introduce a selection bias into the cohort available for study.

Patients have been prospectively followed up and flagged in the Regional Cancer Registry. They were treated in four hospitals in the Merseyside region, two of which have since closed. For this reason, data on recurrence is incomplete, and analysis has only been performed in respect of survival data.

Correspondence: T.G. Cooke, University Department of Surgery, Royal Infirmary, Glasgow, Scotland, G31 2ER, UK.
Received 21 January 1992; and in revised form 27 March 1992.

The close of the study was taken as the 1st of January 1990, at which time the minimum follow up of surviving patients was 93 months.

### Methods

### *DNA measurement*

Ssections of 4 μ and 50 μ were cut from one block of primary tumour from each patient. The thin section was H & E stained to verify the presence of a majority of tumour tissue. Thick sections were processed by the technique of Hedley *et al.* (1983). Sections were wrapped in 50μ nylon mesh and taken through xylene and graded alcohols to water on a tissue processor. The rehydrated tissue was then transferred to plastic tubes and incubated with 0.5% pepsin in 0.9% saline, adjusted to pH 1.5 for 30 min at 37°C, in order to free nuclei. The suspension was washed twice with PBS and then stained with propridium iodide at 30 $\mu g\,ml^{-1}$ in the presence of 100 $\mu g\,ml^{-1}$ RNAase and 0.25% Triton X-100 for 30 min at room temperature. Following this the stained nuclei were resuspended in PBS. Specimens were then analysed on a Coulter Epics Profile II flow cytometer with a 15 mW argon laser emitting at 488 nm, counting 10,000 events at a rate of approximately 100 $s^{-1}$. Red fluorescence was collected using 550 nm long pass dichroic mirror and 610 nm band pass filter. The ploidy histogram was gated on a two dimensional peak versus integral signal histogram in order to eliminate doublets.

### *Assessment of ploidy*

Ploidy histograms were reviewed by a panel of four, and classified as DNA diploid or DNA aneuploid by consensus. In cases of doubt, repeat sections were processed and the two histograms reviewed together. Internal standards were not added to nuclei for flow cytometry, due to the known variation in DNA staining exhibited by paraffin embedded material (Hedley *et al.*, 1985). All presumptively DNA aneuploid histograms showed a substantial population of DNA diploid cells, which acts as an internal standard. Histograms were classified as DNA aneuploid if more than one discrete population was identified. The DNA index was then assigned as the ratio between the mean channel number for the aneuploid G0/G1 peak, and the mean channel for the G0/G1 peak of the normal cell population within the histogram. Diploid tumours have a DNA index of 1.0. Polyploid tumours (those with two or more aneuploid populations of different DNA content) were not assigned a DNA index. Two objective criteria were employed in histogram interpretation.:

(a) histograms were regarded as uninterpretable for ploidy if the half-peak coefficient of variation for the DNA diploid G0/G1 peak was $>10$. Repeat specimens of these blocks were run;

(b) the range of normal for the ratio between G2/M and the corresponding G0/G1 peaks was taken to be 1.85–2.10. Distinct peaks of any size outside this range were regarded as DNA aneuploid if there was a detectable corresponding G2/M peak. Peaks within this range were regarded as representing tetraploid populations if they accounted for $\geqslant 15\%$ of the total histogram events, but only in the presence of a recognisable G2/M peak.

### *Calculation of SPF*

SPF was estimated for each histogram by a single operator using the Cytologics offline analysis software package (Coulter Electronics). Histograms were regarded as interpretable for SPF if the half peak CV of the DNA diploid G0/G1 peak was $\leqslant 8\%$. In the case of DNA aneuploid tumours it was also necessary that some part of the DNA aneuploid population S phase was clearly separate from the usually overlying DNA diploid G2/M peak. Exponential background subtraction was carried out using operator-defined regions either side of the true populations within the histogram. Full peaks were then operator defined, to avoid the inaccuracy of computer identification with its assumption of Gaussian peak shape. The SPF was then calculated using a rectangular model of S phase.

For the purposes of analysis, SPF in DNA diploid and DNA aneuploid tumours have been treated separately. In an DNA aneuploid histogram the specific population under study is separate in the histogram from the DNA diploid population attributable to normal breast cells, non-DNA aneuploid tumour clones, tumour lymphocytes and connective tissue cells. In a DNA diploid histogram these two populations are superimposed, with a consequent 'dilution' of the study population. The SPF as measured in an DNA aneuploid histogram is that of the DNA aneuploid clone alone, whereas the SPF measured in a DNA diploid histogram is that of the total population of cells in the tumour. If we accept that the SPF of non-tumour elements may well be different from that of the tumour, then the two cannot be assumed to be analogous, although many previous reports have assumed that they are.

### *Other data*

Nodal status and tumour size were determined by the hospital pathologist reporting each case. ER assays were performed using the dextran coated charcoal technique, with a cut off of 5 fmol oestradiol mg cytosol protein$^{-1}$. *neu* staining was performed with the 21N polyclonal antibody (a gift of Dr W. Gullick). Tumours were regarded as positive if any areas of tumour membrane staining were seen.

### *Statistical methods*

Survival analyses were carried out to relate survival to ploidy, SPF, lymph node status (positive or negative), tumour size (T1, T2, T3), oestrogen receptor status (positive or negative), and *neu* staining (positive or negative). Patients known to be alive at Jan 1st, 1990 or who were known to have died from causes unrelated to cancer, were treated as censored observations. Univariate analysis was performed using Kaplan-Meier estimates and log rank tests. In light of the differences between SPF as calculated in DNA diploid and DNA aneuploid tumours, the effect of this variable was analysed separately for the two ploidy groups. A combined analysis using a common median was also done, on the grounds that this was the method used in other reports. Multivariate analyses used the Cox proportional hazards regression model, using both forward and backward selection of variables. The tests sought interactions within a model containing the effects of six prognostic variables – ER status, node status, size, *neu* status, ploidy and SPF. SPF was analysed as a binary variable, above or below the median appropriate to the ploidy of that particular tumour.

### Results

### *Patient characteristics*

Histological review indicated that 36 of the blocks examined showed no remaining tumour. The distribution of prognostic factors within the remaining group of 293 patients is indicated in Table I.

### *Ploidy*

Histograms meeting the criteria enunciated in the methods section were obtained in 281 cases (96% of those with tumour-containing blocks). One hundred and seventy-nine (64%) of these were DNA aneuploid, and 102 were DNA diploid. The median CV for the G0/G1 peak in these 281 histograms was 5.6% (interquartile range 4.6–7.0%). The distribution of DNA indices is shown in Figure 1. Univariate analysis of survival stratified by ploidy is shown in Figure 2. There is a survival advantage in favour of DNA diploid tumours of 4% at 5 years and of 3% at 10 years, but this result is not statistically significant. Expressed in terms of a hazards ratio, the relative hazard for patients with DNA aneuploid tumours is 1.20 with 95% confidence limits of 0.81–1.76.

### *SPF*

Estimates of SPF were obtained in 226 cases (80% of those from which ploidy was interpretable). The median value of

**Table I** Characteristics of the study population

| *Variable* | *Subgroups* | *%* |
|---|---|---|
| Nodal status | N0 | 57 |
| | N1 | 43 |
| Tumour size | T1 | 9 |
| | T2 | 71 |
| | T3 | 20 |
| ER status | Pos | 59 |
| | Neg | 41 |
| *neu* status | Pos | 23 |
| | Neg | 77 |
| Menopausal status | Pre | 38 |
| | Post | 62 |

**Figure 1** Frequency histogram showing distribution of DNA indices in aneuploid tumours.

Figure 2 Life table of disease-related mortality for DNA aneuploid and DNA diploid tumours (log rank chi-square = 1.19, 1 df, $P = 0.28$).

Figure 4 Life table of disease-related mortality stratified by SPF below and above the overall median of 7.25% (log rank chi-square = 2.61, 1 df, $P = 0.11$).

SPF was 7.25% overall, 4.5% in DNA diploid tumours and 10.9% in DNA aneuploids. No attempt has been made to compare the distributions of values for DNA diploid and DNA aneuploid tumours statistically, because of the different nature of the measurement in the two types of histogram. Life tables for survival for these populations split at the respective medians are shown in Figure 3. Neither of these analyses show a statistically significant survival effect os SPF. Analysis was also performed within each ploidy group using quartiles of SPF, to assess whether there was an effect restricted to extreme values, but there was no evidence that this was the case. The relative hazard for all tumours with above median SPF, regardless of ploidy, is 1.31 with 95% confidence intervals 0.87–1.98 (Figure 4).

*Multivariate analysis*

This was carried out in the 201 cases where all prognostic variables were available for that tumour. This represents 72% of tumours for which ploidy was measured, and 89% of those for which SPF was available. In a stepwise procedure with all terms starting out of the model, only nodal status is found to be independently prognostic (coefficient 0.67, standard error 0.22, relative hazard 1.96, 95% CI 1.27–3.01). The same result is obtained with a model where all terms start in.

The prognostic effect of ploidy was also analysed in all 255 cases where ploidy, ER status, nodal status, *neu* status and size were available. Adjusting for these factors, no independent prognostic effect for ploidy was observed (coefficient 0.22, standard error 0.20, relative hazard 1.24, 95% CI 0.83–1.87).

Figure 3 Life tables of disease-related mortality for **a**, DNA aneuploid, and **b**, DNA diploid tumours stratified by SPF below or above the median for that tumour type (log rank chi-square = 3.40, 3 df, $P = 0.33$).

### Discussion

We have observed that neither tumour ploidy nor SPF are statistically significant independent prognostic factors in this series of breast cancers. The previous literature upon this subject is confusing. Many reports based upon the flow cytometric study of archival material have been carried out since the technique was developed in 1985. They have been comprehensively reviewed by Merkel and McGuire (1990) and Frierson (1991). Some of these reports are based upon only small numbers of patients, others have studied very diverse groups of patients who have had a variety of different primary treatments, many have used short periods of follow-up for a disease characterised by significant late mortality, and a number have failed to apply multivariate analysis to their findings in order to control for the effect of known prognostic factors within their study group.

Seven reports of which we are aware studied more than 300 patients, followed for a median of at least 5 years, and analysed using multivariate techniques. Cornelisse *et al.* (1987) studied 565 patients with all stages of disease, for up to 10 years, and found DNA aneuploidy to be an independent adverse prognostic factor. In a series of 472 tumours with a minimum of 6 years of follow-up studied by Stål *et al.* (1989), however, ploidy was not an independent prognostic variable. Tumours with a low SPF showed improved survival independent of tumour size, nodal status and ER content, although this was not broken down by ploidy status. They noted that the relationship between disease recurrence and SPF was not significant over the entire follow-up period while controlling for other variables, suggesting that the prognostic value of SPF was reduced by the multivariate analysis.

Toikkanen *et al.* (1989) reported upon the very long term follow-up of 351 patients in whom both ploidy and SPF were measured. Although ploidy predicted strongly for survival at 25 years, this result was not borne out in multivariate analysis. SPF did show independent prognostic significance with low SPF predicting for survival, but SPF was entered as above or below a figure of 7%, chosen on the basis that this provided the greatest distinction between low and high figures. That this figure was selected from the data and then applied back to it, and not validated upon a separate data set weakens the findings in this study. The same criticism can be levelled at Clark et al. (1989), who found ploidy alone to be of independent prognostic significance in a group of 345 patients with node-negative breast cancer, DNA diploid tumours showing an 11% survival advantage at 7 years in univariate analysis which remained significant upon multivariate analysis. They found SPF to be of no additional value in DNA aneuploid patients, but that it was a univariate predictor of survival in DNA diploid patients at cutoffs between high and low SPF of 5.0–9.0%, with a survival advantage to those falling below the cutoff. This effect remained in multivariate analysis using the figure of 6.7%, again not validated upon data from which it was not derived, and furthermore a figure which put 87% of cases into the low SPF group.

Hedley et al. (1987), the originators of the technique for utilising paraffin-embedded material for flow cytometry, performed flow cytometry upon 490 node-positive tumours, with 6 or more years of follow-up. In their series, patients with DNA diploid tumours showed improved survival in univariate analysis, but this effect was no longer evident in multivariate analysis. Tumours with a low SPF showed a survival advantage, using a cutoff very near the median, but this was not evident in multivariate analysis, predominantly due to a strong association with tumour grade.

Kallioniemi et al. (1988) followed 308 patients for 8 years, and found a large univariate survival disadvantage for aneuploid tumours (relative risk 3.0), which was not, however, borne out in multivariate analysis. Ploidy and SPF could be combined to create three prognostic groups which were independent predictors of survival, although once again this relied upon cutoffs determined by examination of the data. Finally, Fisher et al. (1991) have reported upon results from the NSABP-04 trial in 398 patients. This represented only 54% of available tumour blocks, the remainder failing to provide adequate histograms. In this series, ploidy did not predict 10 year survival in univariate analysis. SPF was divided at the median appropriate to that tumour's ploidy, with low SPF tumours having a survival advantage at 10 years of 14%. This result has remained significant in multivariate analysis, although they noted that low SPF tumours still had only a 53% survival at this length of follow-up.

Of the above reports, only two of seven find ploidy alone to be an independent predictor of survival, whilst five of six find SPF or a combination of ploidy and SPF to be such. Our own results are in line with these conclusions, with a non-significant survival difference of only 3% in favour of DNA diploid tumours at 10 years of follow-up despite having over 100 patients still at risk, with a relative hazard of 1.20 to aneuploid tumours. The relative risk observed in multivariate analysis is almost exactly the same, unimproved by the allowance for effects from other variables. In respect of SPF, our results are compatible with the trend toward improved survival in the low SPF tumours, with an observed survival advantage to this group of 5% at 10 years in DNA diploid tumours, and of 11% at that stage in DNA aneuploid tumours. That these results are not statistically significant may represent a type II statistical error. The extent of this potential error can be assessed from the broad confidence intervals for the relative hazards calculated for ploidy and SPF. The relatively low observed level of risk in high SPF tumours may also reflect our decision to divide SPF at the median rather than at a level chosen from the data, a process which however is open to criticism as stated already.

The assignment of DNA index and SPF are subject to a number of technical pitfalls. We know from cytogenetic studies that all tumours contain abnormal quantities of DNA. The DNA index at which a tumour becomes interpretable as DNA aneuploid is arbitrary, depending upon the quality of the flow cytometry. Tumours with grossly abnormal DNA content may also be difficult to define as DNA aneuploid, in situations where the number of DNA aneuploid cells is low, in which case the DNA aneuploid peak may be obscured by background counts, or by the DNA diploid G2/M peak. It is also presumed that tumours are consistent in their ploidy, that is that there is no variation in DNA content between different areas of tumour. However, there is good evidence of spacial heterogeneity of ploidy within the literature (Fuhr et al., 1991; Kallioniemi, 1988). The calculation of SPF by the use of computer models presents further problems. Firstly, SPF is not interpretable in all tumours. Computer models of DNA histograms assume that all peaks are Gaussian, which is observably not always so. It is also necessary to substract background counts from the histogram which introduces an element of subjectivity into the process of SPF estimation.

In conclusion, we have demonstrated no statistically significant independent effect upon long-term survival in breast cancer for either tumour ploidy or S-phase fraction. The technical factors which limit the accuracy and reproducibility of this information may at least partly explain these findings, which upon critical analysis are borne out by previous studies. The potential of flow cytometric indices of DNA as prognostic factors in breast cancer is limited in our hands.

### References

CLARK, G.M., DRESSLER, L.G., OWENS, M.A., POUNDS, G., OLDAKER, T. & MCGUIRE, W.L. (1989). Prediction of relapse or survival in patients with node-negative breast cancer by DNA flow cytometry. *New Engl. J. Med.*, **320**, 627–633.

CORNELISSE, C., VAN DER VELDE, C., CASPERS, R., MOOLENAAR, A. & HERMANS, J. (1987). DNA ploidy and survival in breast cancer patients. *Cytometry*, **8**, 225–234.

FISHER, B., GUNDUZ, N., COSTANTINO, P.H., FISHER, E.R., REDMOND, C., MAMOUNAS, E.P. & SIDERITS, R. (1991). DNA flow cytometric analysis of primary operable breast cancer. Relatio of ploidy and S-phase fraction to outcome of patients in NSABP B-04. *Cancer*, **68**, 1465–1475.

FRIERSON, H.F. (1991). Ploidy analysis and S-phase fraction determination by flow cytometry of invasive adenocarcinomas of the breast. *Amer. J. Surg. Path.*, **15**, 358–367.

FUHR, J.E., FRYE, A., KATTINE, A.A. & VAN METER, S. (1991). Flow cytometric determination of breast tumour heterogeneity. *Cancer*, **67**, 1401–1405.

HEDLEY, D.W., FREIDLANDER, M.L., TAYLOR, I.W., RUGG, C.A. & MUSGROVE, E.A. (1983). Method for analysis of cellular DNA content of paraffin-embedded tissue. *J. Histochem. Cytochem.*, **31**, 1333–1335.

HEDLEY, D.W., FREIDLANDER, M.L. & TAYLOR, I.W. (1985). Applications of DNA flow cytometry to paraffin-embedded archival material for the study of DNA aneuploidy and its clinical significance. *Cytometry*, **6**, 327–333.

HEDLEY, D.W., RUGG, C. & GELBER, R. (1987). Association of DNA index and S-phase fraction with prognosis of node positive early breast cancer. *Cancer Res.*, **47**, 4729–4735.

KALLIONIEMI, O.-P. (1988). Comparison of fresh and paraffin-embedded tissue as starting material for DNA flow cytometry and evaluation of intratumour heterogeneity. *Cytometry*, **9**, 164–169.

KALLIONIEMI, O.-P., BLANCO, G., ALAVAIKKO, M. & 8 others. (1988). Improving the prognostic value of DNA flow cytometry in breast cancer by combining DNA index and S-phase fraction. A proposed classification of DNA histograms in breast cancer. *Cancer*, **62**, 2183–2190.

MERKEL, D.E. & MCGUIRE, W.L. (1990). Ploidy, proliferative activity and prognosis. DNA flow cytometry of solid tumours. *Cancer*, **65**, 1194–1205.

STÅL, O., WINGREN, S., CARSTENSEN, J., RUTQVIST, L.E., SLOOG, L., KLINTENBERG, C. & NORDENSKJOLD, B. (1989). Prognostic value of DNA ploidy and S-phase fraction in relation to estrogen receptor content and clinicopathological variables in primary breast cancer. *Eur. J. Cancer Clin. Oncol.*, **25**, 301–309.

TOIKKANEN, S., JOENSUU, H. & KLEMI, P. (1989). The prognostic significance of nuclear DNA content in invasive breast cancer – a study with long-term follow-up. *Br. J. Cancer*, **60**, 693–700.